# The impact of multifactorial factors on the Quality of Life of Behçet's patients over 10 years

**DOI:** 10.3389/fmed.2022.996571

**Published:** 2022-12-05

**Authors:** Amal A. Senusi, John Mather, Dennis Ola, Lesley A. Bergmeier, Bindi Gokani, Farida Fortune

**Affiliations:** ^1^Centre for Oral Immunobiology and Regenerative Medicine, Barts and The London School of Medicine and Dentistry, Queen Mary University of London, London, United Kingdom; ^2^Royal London Hospital, London Behçet's Centre, Barts Health London, London, United Kingdom; ^3^Behçet's Patients Support, Behçet's Patients Centres, London, United Kingdom

**Keywords:** Behçet's syndrome symptoms, Quality of Life, EQ-5D measure, social and psychological wellbeing in Behçet's patients, autoinflammatory conditions, socio-demographics in BD

## Abstract

**Objective:**

This study analyses the 2020 survey and reviews the 2009, 2014 surveys to ascertain which Behçet's symptoms, personal and family status, patients' lifestyle, and work-related outcomes impacted on Health-Related Quality of Life (HRQoL).

**Methods:**

Four hundred and fifty-nine Behçet's patients submitted an online survey/questionnaire. Patients provided information on socio-demographic characteristics, disease duration, historical and current symptoms, systemic and topical medication, health related lifestyle, work-related outcomes regarding employment status and claiming benefits and Quality of Life (QoL) measured by EQ-5D index.

**Results:**

Four hundred and nineteen patients met the inclusion criteria, and 371 who had full data (Males: Females: Others = 84:285:2, mean–age = 41.1 ± 23.3:38 ± 13.2:40 ± 5). The main symptoms associated with patients seeking medical care were mouth ulcers 30% and genital ulcers 23%, joint 14%, and eye problems 9%. The EQ-5D index for 2009, 2014, 2020 was (mean ± SD); 0.47 ± 0.38, 0.42 ± 0.37, 0.34 ± 0.40, respectively, *p* < 0.05. 2020 patients had the worst values of the five domains compared to 2014 and 2009. Interestingly, mobility value was the same over the 10 years of monitoring patients. Behçet's syndrome (BS) symptoms that had significant negative impact on QoL were; 2009 (arthropathy, neurological problems, pathergy reaction, and stomach/bowel symptoms), 2014 (arthropathy, headache, neurological problems, pathergy reaction, and skin lesions), 2020 (arthropathy, neurological problems, and stomach/bowel symptoms). The 2014 and 2020 surveys reported the QoL is significantly better in patients on immunosuppressant, who did sport, continued in employment and not receiving benefits.

**Conclusion:**

Joints and neurological symptoms are the main symptoms which had negative impact on BS patients over the 10 years, sociodemographic (gender, age, marital, and education status), lifestyle (medication, cannabis, drinking wine, and regular exercise), employment status (employee and no career change), and accessing benefits (never claim benefit) had significant influence on patients' HRQoL.

## Introduction

Behçet's syndrome (BS) is a chronic autoinflammatory systemic disorder which impacts on patient's Quality of Life (QoL) (1, 2). The symptoms and overall effect of the disease pattern are variable and changes as the condition progresses. It is important to recognise and understand the impact of BS on patients' Health-Related Quality of Life (HRQoL) and continually moderate the ongoing management.

Quality of Life is a broad multidimensional concept with numerous domains. Although health is one of the domains of QoL, other key domains; family status, jobs, housing, education-level, lifestyle, psychological, and mental health status adding to the complexity of its measurement. The UK National Health Service review working paper on medical audit ascertained that QoL is an essential part audit procedures (1).

The majority of previous studies in Behçet's either reviewed the relationship between the disease activity and QoL (2–5), or compared the QoL of BS patients who presented with and without joints involvement, or compared QoL of BS patients with rheumatoid arthritis (RA), and eye involvements in comparison to healthy control (6), or explored the differential or cumulative impact of multiple symptoms on QoL (7). To our knowledge this study is unique in the continued reassessment the QoL of BS patients from 2009, 2014 to 2020. The importance of this study to compare BS patients EQ-5D index every 5 years and evaluate the many factors that have impact on patients' QoL.

Health-Related Quality of Life is a legitimate construct for evaluating patient treatment, side effects and is significantly associated with patients' survival regardless of the time of disease assessment. It is used as a tool to predict mortality (7–9). Analysis of HRQoL data can identify subcategory factors that influence health, and subsequently assist in directing interventions to improve their condition, preventing more serious health consequences. Interpretation and publication of data raises awareness for resources for unmet needs. It assists in identifying requirements for health-policies, legislation, and guides strategic development plans.

Using data from HRQoL of BS patients' surveys carried out in 2009 and 2014 and comparing it with 2020 data, we demonstrate significant health-care interventions in this population cohort, particularly in those using the three commissioned Behçet's Centres. Notable among these are changes to medication and the treatment of BS. Others include social and psychological support, oral health promotion, and healthy lifestyle including dietary habits, alcohol, and smoking/cannabis use.

## Materials and methods

Four hundred and fifty-nine BS patients were diagnosed according to the International Criteria of Behçet's Disease (ICBD 2014) who submitted the online survey/questionnaires devised together with the Society's Behçet's Patients Centres (BPC)-UK, the links are; https://www.surveygizmo.eu/s3/90209097/2020-BEHCETS-QoL-SURVEY, http://sgiz.eu/s3/2020-BEHCETS-Qol-SURVEY, https://behcetspatients.org.uk/, and https://apps.talktalk.co.uk/appsuite/. The survey was available online from March 2020 through to August 2020. The survey promoted via the news section of the websites of Behçet's UK, Behçet's Patients Support, and in the quarterly newsletter of Behçet's UK. It was also promoted in social media channels of Behçet's UK (Facebook and Twitter).

Participants press the “agree button” to indicate that they had read the instructions and information sheet e.g., are voluntarily agreeing to participate, and at least 18 years old. A confirmed diagnosis of BS was a prerequisite to enrol and complete the on-line survey. Fifteen to twenty minutes was the average duration to complete this online survey. Exclusions were patients with difficulty understanding English, replies with minimal information. One reminder was sent to increase the response rate.

This study applied a cross-sectional survey design, using the same questionnaire that was used in the 2009 and 2014 studies. However, 2020 survey was designed as a digital/online form (digitisation J. Mather). Ethical approval was obtained from the Queen Mary Research Ethics Committee; City Research Ethical Committee (COREC) approved study “Immune-regulation at the mucosal barrier” (P/03/122) at Barts Health NHS Trust in full compliance with the Helsinki Declaration (10).

The survey questionnaire gathered information on participants' socio-demographic; gender, age, country of birth, ethnicity, height, weight, marital status, qualification, employment status, access to internet, homeowner, and education level. Disease characteristics, BS triggers, age first symptoms present, order of symptoms, and age of diagnosis, professional making diagnosis, current symptoms, and symptom control using topical and systemic medication, symptoms impact on QoL.

Participants chose current BS symptoms from a list of common BS manifestations; mouth ulcers, genital ulcers, skin lesions, fatigue, arthropathy, gastrointestinal symptoms, ocular problems, pathergy reaction, headache, and neurological symptoms. Health related lifestyle: information on tobacco use; cigarettes, cigar, pipes, cannabis and snuff, age when started and stopped, alcohol amount, variety, and whether interested in quitting alcohol consumption. Food; types, portions, content, and preparation, whether food exacerbated symptoms. Exercise for more than 30 min/day were included. Oral hygiene advocacy: use of dental floss, interdental/toothpicks/wood-sticks, dental disclosing-tablets, dental chewing-gum, mouthwash, inter-space brush, e-toothbrush, lauryl sulphate free or smokers' toothpaste. Work-related outcomes: BS effects on work; sick leave, and motivation to keep patients in the workplace. Career changes; reduced working hours, changed to a less stressful job, had to give-up work. Stated benefit/support; Personal Independence Payment (PIP), Employment and Support Allowance (ESA), and Universal Credit (UC).

The patients' HRQoL was measured using a valid and reliable self-completed EQ-5D-L3 questionnaire (7, 11), consisting of five questions encompassing the health domains; mobility, self-care, usual activity, pain, and anxiety/depression. Participants were asked to select their level of problem in each domain from three options: 1 (no problems), 2 (moderate problems), and 3 (extreme problems). The choices resulted in a five-digit score for each participant which reflects a unique health state, e.g., state 11223 indicates no problems with mobility and self-care, some problems with performing usual activities, moderate pain or discomfort and extreme anxiety or depression, while state 11111 indicates no problems in any of the five domains.

A tool “EQ-5D Index Value Calculator” by R package (https://shiny.rstudio.com/) was used to calculate the score of 419 observations. The value was set for the UK (https://euroqol.org/) to provide QoL index/patients. The EQ-VAS was recorded for each participant in scale of 100 (the best health) to 0 (the worst health).

### Statistical analysis

The data was analysed using SPSS Statistics software (version 28; IBM Corporation, USA) and R-studio (https://shiny.rstudio.com/). Number of symptoms reported by each participant, ranging between 0 and 10. Descriptive analysis (mean ± SD), and multiple regression analysis was performed in this study (*R*^2^ is a statistical measure of how close the data are to the fitted regression line). The higher the beta value the greater the impact of the predictor variable on the dependent variable. Independent *t*-test and one-way ANOVA tests were used to determine if there are any significant differences between the variables with EQ-5D index. Fisher's Least Significant Difference (LSD) test was included. *p* < 0.05 was accepted to be a significant result.

## Results

A total of 419 BS patients met the inclusion criteria and agreed to participate, 371 had their EQ-5D score (Males:Females:Others = 84:285:2; mean age ± SD = 41.1 ± 23.3:38 ± 13.2:40 ± 5), 98.6% of patients had regular access to the internet. Sixty-nine percent of respondents were women, 21% men, and two patients were LGBT (lesbian, gay, bisexual, transgender). Eighty-six percent were white-British, 4% white-European, and 10% were black and minority ethnic groups. Supplementary Figure S1 demonstrates the geographic distribution of the cohort which seems it follows the Silk-Route map. International patients: one patient from each country (Australia, Germany, Malta, Czech Republic, Belgium, and China), and two patients from USA were excluded from the map.

**Figure 1:**
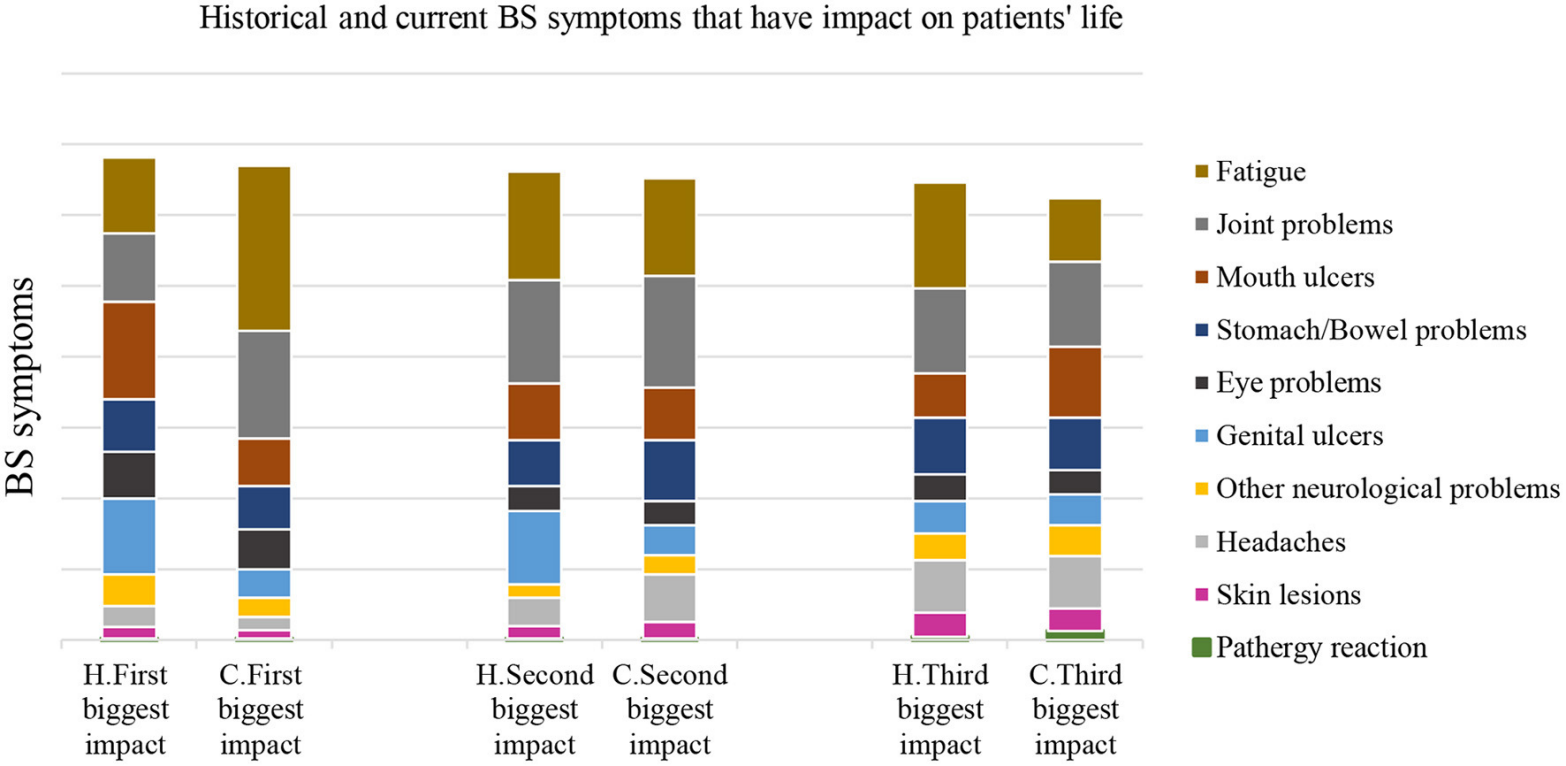
BS patients and their answer about the most historical (H) and current (C) symptoms that have impact on their life of 2020 survey. The historical symptom that had a negative impact on patients HRQoL, were ranked as follows; mouth ulcers that had a high impact on their QoL. The 2^nd^ and 3^rd^ ranking was mainly fatigue and joints problems, respectively. However, the current symptoms that had negative impact on their HRQoL; 1^st^, 2^nd^, and 3^rd^ were fatigue, joint problems, and then oral ulceration.

The participants' age-range; (18–34 years) 14%, (35–54 years) 36%, and (>55 years) 28% (Table 1). Patients were referred by; rheumatologist 59%, ophthalmologist 8%, General Medical Practitioners (GMPs) and immunologist 6% each, dermatologist and neurologist 5% each, and oral specialist 4%. Smaller percentages were referred by sexual health clinicians, general dental practitioners, gynaecologist, gastroenterologist, and paediatrician (Supplementary Figure S2). The main BS historical symptoms; oral ulcers 99.4%, arthropathy 90%, fatigue 88%, genital ulcers 84%, skin lesions 79%, stomach problems 75%, headaches 72%, eye problems 64%. However, BS current symptoms; fatigue 92%, followed by mouth ulcer 85%, joint problems 84%, and headaches 66% (Figure 1).

**Table 1:** Socio-demographic, lifestyle, career, and social benefits characteristics of BS patients and their EQ-5D scores (*n* = 371).

**Variables**	**Pt. no**.	**EQ-5D index (Mean ±SD)**	***P*-value**
**Gender**			
Men	84	0.58 ± 0.33	0.035*
Women	285	0.48 ± 0.26	
Other	2	0.19	
Patients did not fill-in the EQ-5D elements	48	**–**	**–**
**Age in years**			
**Age range in three groups**			
G1 = 18–34	59	0.58 ± 0.25	
G2 = 35–54	151	0.46 ± 0.29	G2 vs. G1 = 0.005*
G3 = 55–73	117	0.51 ± 0.29	
**Marital situation**			
G1 = Single	71	0.49 ± 0.27	G4 vs. G1 = 0.009*
G2 = Married (with children)	143	0.50 ± 0.29	G4 vs. G2 = 0.002*
G3 = Married (without children)	28	0.63 ± 0.22	G4 vs. G3 = 0.001*
G4 = Divorced (with children)	38	0.35 ± 0.22	
G5 = Divorced (without children)	8	0.52 ± 0.26	
G6 = Widowed	17	0.43 ± 0.30	
**Ethnicity**			
G1 = White British	288	0.36 ± 0.40	
G2 = White non-British	29	0.38 ± 0.21	G1 vs. G4 = 0.019*
G3 = Asian	10	0.35 ± 0.41	
G4 = Others	15	0.34 ± 0.39	
**Education level**			
G1 = No education/degree	20	0.20 ± 0.37	G1 vs. G5 = 0.017*
G2 = Secondary school (O-level/GCSE)	84	0.31 ± 0.41	G1 vs. G6 = 0.021*
G3 = A level	48	0.27 ± 0.37	
G4 = Technical qualifications	53	0.29 ± 0.42	G5 vs. G2 = 0.040*
G5 = First degree (university)	72	0.43 ± 0.391	G5 vs. G4 = 0.046*
G6 = Higher degree (post-graduate)	46	0.54 ± 0.37	
**Fruit and vegetable 5 portions/day**			
Yes	147	0.52 ± 0.29	0.285
No	176	0.49 ± 0.28	
**Four different varieties of fruit each week**			
Yes	193	0.52 ± 0.29	0.386
No	127	0.49 ± 0.27	
**Four different varieties of vegetables each week**			
Yes	262	0.51 ± 0.29	0.422
No	63	0.48 ± 0.27	
**Oral hygiene factors**			
**Dental floss**			
Yes	153	0.47 ± 0.27	0.06
No	182	0.53 ± 0.28	
**Interdens/Toothpicks/Woodsticks**			
Yes	103	0.49 ± 0.25	0.522
No	232	0.51 ± 0.29	
**Dental disclosing tablets**			
Yes	5	0.37 ± 0.18	0.182
No	330	0.50 ± 0.28	
**Dental chewing gum**			
Yes	18	0.39 ± 0.30	0.150
No	317	0.51 ± 0.28	
**Mouthwash**			
Yes	215	0.44 ± 0.27	0.001*
No	120	0.60 ± 0.27	
**Interspace brush**			
Yes	70	0.48 ± 0.28	0.533
No	265	0.51 ± 0.28	
**Electric toothbrush**			
Yes	220	0.49 ± 0.28	0.690
No	115	0.511 ± 0.27	
**Toothpaste without (SLS)**			
Yes	92	0.48 ± 0.29	0.513
No	243	0.51 ± 0.27	
**Other**			
Yes	34	0.44 ± 0.25	0.025*
No	301	0.51 ± 0.28	
**Tobacco use**			
Non-smoker	182	0.53 ± 0.23	
Current smoker	30	0.42 ± 0.24	0.071
Smoker (in the past)	109	0.48 ± 0.28	
**Cannabis use**			
Yes	45	0.42 ± 0.30	0.045*
No	274	0.36 ± 0.40	
**Drinking alcohol**			
**Number of 1/2 pints-Beer or Cider/W**			
One and more	16	0.57 ± 0.36	0.27
No	235	0.49 ± 0.28	
**Number of singles-Spirits or Liqueurs/W**			
One and more	72	0.51 ± 0.27	0.29
No	192	0.47 ± 0.28	
**Number of glasses-Wine/W**			
One and more	97	0.69 ± 0.18	0.001*
No	171	0.46 ± 0.29	
**Exercise for 30 min a day**			
G1 = Never	121	0.37 ± 0.26	
G2 = Once	29	0.52 ± 0.29	G1 vs. all other
G3 = Twice	49	0.59 ± 0.29	groups ***P*** **<** 0.05*
G4 = Three times	49	0.55 ± 0.26	
G5 = Four times	23	0.64 ± 0.23	
G6 = Five times	26	0.61 ± 0.25	
G7 = Six times	11	0.68 ± 0.21	
G8 = Seven times	22	0.63 ± 0.25	
**BS medication**			
G1 = Immunosuppressants (Azathioprine, Tacrolimus, Cyclosporin, and MMF)			
Yes	142	0.51 ± 0.28	0.629
No	193	0.49 ± 0.28	
G2 = Chemotherapy drugs (Chlorambucil, Methotrexate, and Cyclophosphamide)			
Yes	32	0.37 ± 0.25	0.003*
No	303	0.52 ± 0.28	
G3 = Biological agents (Interferon Alpha, Infliximab, Adalimumab, and Etanercept)			
Yes	82	0.46 ± 0.29	0.126
No	253	0.51 ± 0.27	
G4 = Steroids (orally or infusion)			
Yes	163	0.45 ± 0.26	0.001*
No	172	0.55 ± 0.29	
G5 = Topical Treatments (drops, gel, cream, and mouthwash)			
Yes	204	0.44 ± 0.27	0.001*
No	131	0.60 ± 0.26	G1 vs. G2 = 0.01*
			G1 vs. G3 = 0.20
			G1 vs. G4 = 0.053
			G2 vs. G3 = 0.125
**Career changes**			
G1 = No	99	0.64 ± 0.27	G4 vs. G1 = 0.001*
G2 = Yes (reduced my working hours)	54	0.58 ± 0.22	G4 vs. G2 = 0.001*
G3 = Yes (changed to a less stressful job)	25	0.58 ± 0.24	G4 vs. G3 = 0.001*
G4 = Yes (have had to give up work)	125	0.34 ± 0.23	
**Benefit claim**			
G1 = Never claim benefit	126	0.66 ± 0.27	G1 vs. G3 = 0.001*
G2 = Not now but have received benefit in the past	9	0.66 ± 0.25	
G3 = Yes and been successful	152	0.37 ± 0.23	
**Claiming PIP**			
Yes (receive PIP)	128	0.35 ± 0.22	0.001*
No (unsuccessful in claiming PIP)	207	0.59 ± 0.27	
**Claiming ESA**			
Yes (receive ESA)	60	0.33 ± 0.23	0.001*
No (unsuccessful in claiming ESA)	275	0.54 ± 0.28	
**Claiming UC**			
Yes (receive UC)	16	0.41 ± 0.32	0.278
No (unsuccessful in claiming UC)	319	0.50 ± 0.28	

G, group; Pt. No., patients' number; W, week; MMF, Mycophenolate Mofetil; SLS, Sodium lauryl sulphate. Personal Independence Payment (PIP) is a benefit for people who may need help with daily activities or getting around because of a long-term illness or disability; Employment and Support Allowance (ESA) is a benefit for people who cannot work because of an illness or disability; Universal Credit (UC) is a payment to help with your living costs. It's paid monthly- or twice a month for some people in Scotland (https://www.gov.uk/).

*P-values are significant.

Bold values are statistically significant values.

The symptoms that led patients seeking medical advices were mouth ulcers 30%, genital ulcers 23%, joint problems 14%, and eye symptoms 9%. Forty-nine percent were used steroid medication, 44% taking immunomodulatory medication, and 24% biological medication (Table 1).

Almost 72% of patients with moderate-extreme anxiety/depression (scale of 1–3), 49% had washing/dressing trouble, and 84% had problems performing common daily activities. Twenty-eight percent stopped working because of BS resulting in low QoL (EQ-5D score: mean ± SD; 0.34 ± 0.23), and 55% claimed social benefits. In terms of oral hygiene, 71% brushing twice daily, 68% used an e-toothbrush, 67% used mouthwash/day, and 58% visited dentists within the last 6 months.

Fifty-seven percent had never smoked, and 34% had smoked in the past with EQ-5D score: mean ± SD; 0.53 ± 0.23 and 0.48 ± 0.28; *p* = 0.09, respectively. Patients who were current smokers had lowest QoL: mean ± SD; 0.42 ± 0.24. The data indicates that 45 patients used cannabis with a score of EQ-5D (mean ± SD: 0.42 ± 0.30). Thirty-six percent had tattoos which linked to BS symptoms (12, 13).

### EQ-5D index and patients' gender, age marital status, and education level of 2020 survey

The QoL of BS males were significantly higher than females, *p*-value = 0.035. The QoL of 18–34 years group were significantly higher compared to 35–54 year group; *P* = 0.005 (Table 1). The statistical comparison between the subgroups of family status and QoL of BS patients was significant (*P* = 0.007); divorced patients (with children) had significantly lower QoL than those who were: single, married with children, or married without children, *P* = 0.009, 0.002, and 0.001; respectively (Table 1).

There were significant differences of QoL between subgroups of patients with different education levels (*P* = 0.043); no-education/no-degree group had significantly worse QoL than those with first and higher degree; *P* = 0.017, and 0.021, respectively. Patients with post-graduate degrees had the best QoL (Table 1).

### EQ-5D index and patients' lifestyle of 2020 survey (oral hygiene, tobacco smoking, drinking alcohol, paan, cannabis use, diet, and exercise)

Table 1 shows that patients who used mouthwash and other dental hygiene products had significantly less QoL than who not using these products (*p* = 0.001, and *p* = 0.025, respectively). Patients who never smoked had higher QoL (mean ± SD; 0.53 ± 0.23) than current smokers, or smoked in the past, with the differences just failing to be significant (*P* = 0.071).

Forty-five patients using cannabis had higher QoL scores (mean ± SD; 0.42 ± 0.30) than who were not (mean ± SD; 0.36 ± 0.40); *p* = 0.045. Patients who drank one glass of wine or more, had higher QoL than who did not drink wine, *p* = 0.001 (Table 1). The analysis of diet was complex as the consumption of fruit and vegetables appeared to have no impact on outcomes. Two hundred and fifty patients avoided fizzy drinks, and 35 were vegetarians.

Patients who never exercised had significantly worse QoL compared to other groups who did exercise daily, *P*-values were < 0.05 (Table 1).

### EQ-5D index and patients' medication of 2020 survey

Table 1 shows that patients not taking chemotherapy, steroids, or topical treatment had significantly higher QoL than who used these medications (*p* = 0.003, 0.001, and 0.001, respectively). However, there was no significant difference between the QoL of patients taking immunosuppressant or biologics, compared to those not taking these medications. The QoL of patients who were using immunosuppressant was significantly two times better than patients on chemotherapy; *p* = 0.01. The QoL of immunosuppressant patients compared to steroid patients just failed to be significant; *p* = 0.053.

### EQ-5D index and patients' employment status, changing career, and claiming benefits results of BS of 2020 survey

Patients who discontinue working due BS have significantly worse QoL compared to patients who remained in employment (did not changed their career, reduce working hours, or changed to less stressful job); *P* = 0.001 between the groups.

There were significant differences (*P*-values were ≤ 0.001) in the EQ-5D scores between subgroups of patient employment status. The working groups of volunteering workers, students, the full-time and part-time, had significantly higher QoL than those unemployed due to health problems (Table 1).

Table 1 summary of multiple comparison analysis between claiming benefits subgroups; never claiming benefits, claimed in the past, and claimed recently, was significant (*P* = 0.001). Further analysis showed that BS patients who were not receiving any benefits/never claimed benefit in the past had significantly better QoL than those who were receiving benefits (*p* = 0.001).

### Comparing EQ-5D index domains: Mobility (MO), self-care (SC), usual activity (UA), pain/discomfort (PD), and anxiety/depression (AD) of 2009, 2014, and 2020 survey

**Figure 2:**
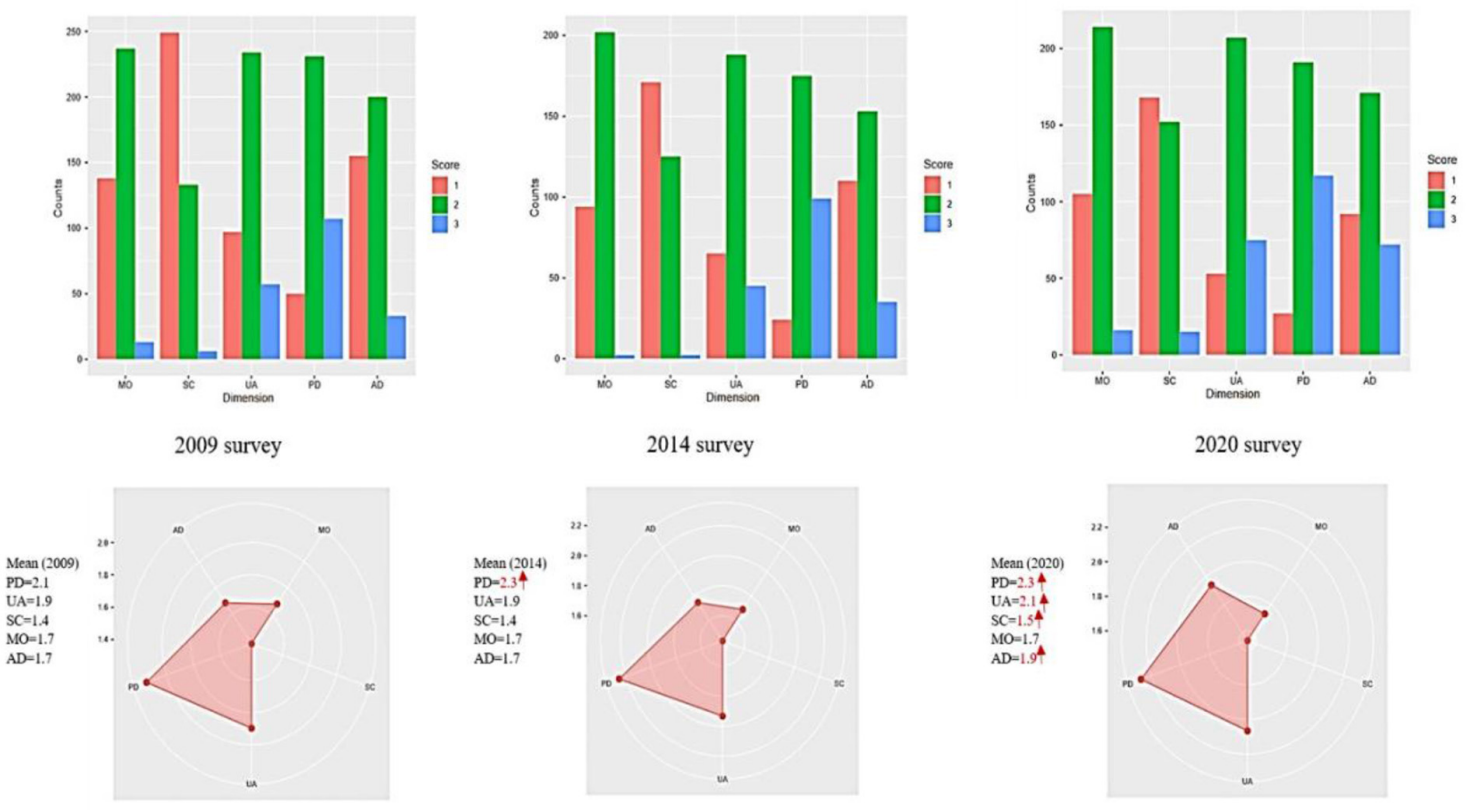
The mean values of EQ-5D 3L domains: mobility (MO), self-care (SC), usual activity (UA), pain and discomfort (PD), and anxiety/depression (AD), and the number of patients' responses in 2009, 2014, and 2020 surveys. 3L (three levels); 1 = no problem, 2 = some problems, and 3 = severe problems.

**Figure 3:**
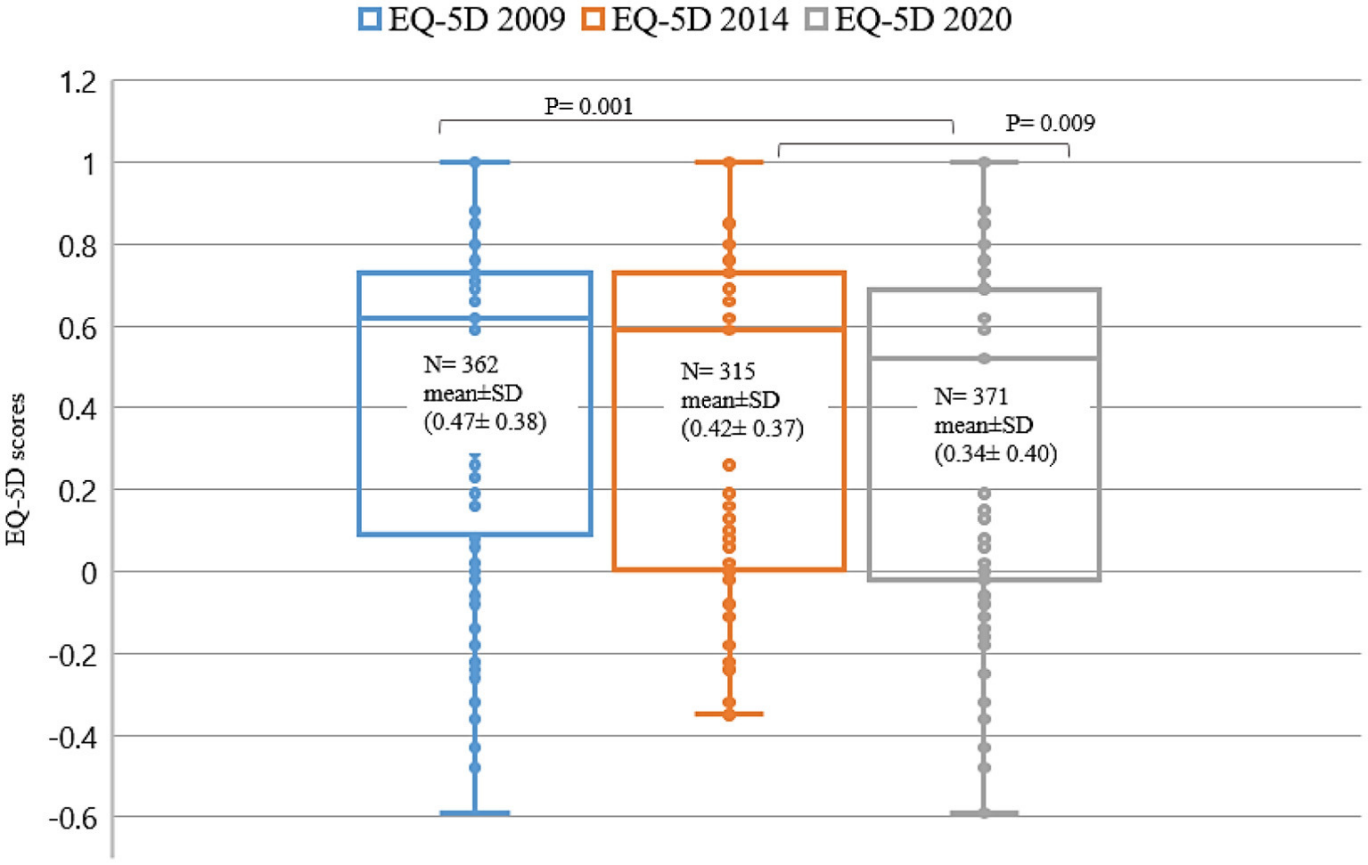
The differences of EQ-5D index for 2009, 2014, and 2020 patients. The EQ-5D (means ± SD) indices for 2009, 2014, and 2020, were; 0.47 ± 0.38, 0.42 ± 0.37, and 0.34 ± 0.40, respectively; *p* < 0.05.

The EQ-5D domain mean of 2009, 2014, and 2020 survey are presented in radar plots; Figure 2, demonstrate that 2020 patients had the worst values of the five domains compared to 2014 and 2009. Interestingly, MO value was the same over the 10 years of monitoring patients. Figure 3 shows difference between EQ-5D index (mean ± SD) of 2009, 2014, and 2020; 0.47 ± 0.38, 0.42 ± 0.37, 0.34 ± 0.40; respectively. The QoL of 2020 BS patients was significantly less than 2009 and 2014 patients; *P* = 0.001, and 0.009, respectively.

### The impact of BS symptoms on patients' QoL of 2020 survey and compared to 2009 and 2014 surveys

**Figure 4:**
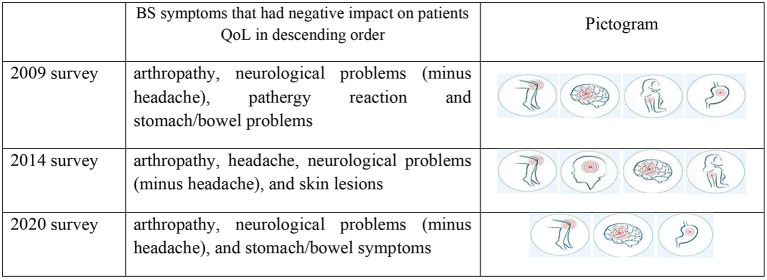
Pictogram shows the descending order of main BS symptoms that have negative impact on patients QoL. Both arthropathy and neurological problems presented in three different time points as a main problems that effect on patients' HRQoL. Source: https://st2.depositphotos.com/1411892/6582/v/950/depositphotos_65828131-stock-illustration-body-pain.jpg.

The model (dependent variable; EQ-5D scores, and independent variable; BS symptoms) was tested using regression analysis (*R* = 0.39, *R*^2^ = 0.143 *R*^2^adj = 0.12) of 2020 survey. The main symptoms that negatively impacted on patients' HRQoL in 2020 survey were; arthropathy, neurological problems, and stomach/bowel symptoms in decreasing order (*P-*values were; 0.022, 0.001, and 0.001 and the coefficients values were; −0.128, −0.123, and −0.111, respectively) (Table 2). In 2014 survey were; arthropathy, headache, neurological features, pathergy reaction, and skin lesions had the significant impacted on the HRQoL (coefficients value were −0.336, −0.227, −0.135, −0.119, and −0.107; respectively). Similarly in the 2009 survey; arthropathy, neurological problems, pathergy reaction, and stomach/bowel problems (coefficients value −0.15, −0.13, −0.11, and −0.18; respectively) (Figure 4).

**Table 2:** The impact of BS symptoms on EQ-5D index of patients who answered 2020 survey.

	**No**.	**Unstandardized coefficients**		**Standardised coefficients**	***P*-value**
		***B* (95% CI)**	**Std. error**	**Beta**	
**Model (dependent variable, EQ-5D scores and independent variables, Behçet's symptoms list)**					
Mouth ulcers	332	−0.014 (−0.332,0.304)	0.162	−0.005	0.931
Genital ulcers	282	0.022 (−0.062,0.106)	0.043	0.029	0.604
Skin lesions	266	−0.008 (−0.084,0.067)	0.038	−0.012	0.833
Fatigue	307	0.063 (−0.058,0.184)	0.062	0.061	0.309
Arthropathy	301	−0.128 (−0.238, −0.019)	0.056	−0.137	0.022*
Stomach/Bowel problems	207	−0.111 (−0.174, −0.048)	0.032	−0.190	0.001*
Eye problems	216	0.012 (−0.049,0.074)	0.031	0.021	0.696
Pathergy reaction	115	−0.027 (−0.092,0.038)	0.033	−0.046	0.411
Headaches	240	−0.037 (−0.109,0.0340)	0.036	−0.060	0.302
Other neurological problems^‡^	141	−0.123 (−0.188, −0.057)	0.033	−0.214	0.001*

^‡^Other neurological problems such as cerebral venous sinus thrombosis (CVST) (extra-axial NBS), CNS involvement (intra-axial NBS), arterial NBS, neuro-psycho-Behçet's syndrome, peripheral nervous system (PNS) involvement, and subclinical NBS.

*P-values are significant.

Bold values are statistically significant values.

## Discussion

Judicious reporting of symptom progress and severity, health outcomes, and the HRQoL information by patients will enhance the understanding of evolution of BS over time. This in turn will assist the planning of constructive interventions. Psychological support should remain an important component of management with 72% of our cohort suffering from moderate or extreme anxiety and depression.

This project provided the opportunity to retrospectively review surveys carried out in 2009 and 2014 compared to a 2020 survey, and demonstrates the associations between BS symptoms, personal/family status, patients' lifestyle, claiming benefits, and employment status with HRQoL scores.

The strategy of collecting data for the 2020 BS survey was changed to an on-line questionnaire because of SARS-CoV-2 pandemic, unlike the previous two surveys where postal questionnaires were used, and more questions were added to the 2020 survey. These included questions on “home ownership” (63% of respondents answered yes), and “regular access to the internet” (about 99% of respondents had access). The response rate of 2020 survey and 2014 and 2009 showed cohort differences between sexes with respondents in favour of the females as women are more compliant than men in contributing to such studies (14). The ethnicity of the respondents in this study was mainly white-British, and white-European. Only 10% were black and minority ethnic groups which is smaller our patient cohort although we encouraged participation.

In 2020 survey the BS symptoms activity was linked primarily to stress acting as the main trigger, followed by fatigue, then cold or other infections, and finally seasonal changes. Rheumatologists, ophthalmologists, and GMPs were the main referring physicians. Behçet's symptoms historical symptoms were ranked as follows; oral ulcers ranked the greatest negative impact on QoL. The 2^nd^ and 3^rd^ selections were mainly fatigue and joints problems, respectively. However, the current symptoms; 1^st^, 2^nd^, and 3^rd^ were fatigue, joint problems, and then oral ulceration (Figure 1). 2014 survey reported that 85.4% of patients suffered from fatigue; however, the 2020 survey about 92% suffered from fatigue.

Regression modelling demonstrated that the BS symptoms that impacted negatively on the QoL in 2009 were; arthropathy, neurological problems, pathergy reaction, and stomach/bowel symptoms, whereas, in 2014; arthropathy, headache, neurological problems, pathergy reaction, and skin lesions. In 2020 survey were; arthropathy, neurological problems, followed by stomach/bowel problems. This indicates constant review permits provision of suitable interventions to ameliorate the burden of symptoms which impact on QoL and general wellbeing (7, 11).

These BS surveys have allowed the gathering of data to assist in providing evidence-based advice to patients and patient advocates, as well-moderating use of medication used to alleviate their symptoms. For example, the use of mouthwashes (TMW and subsequently Triorasol) (15) are proven to be beneficial in reducing BS oral symptoms. TMW is readily available to all our BS patient at an affordable price (given that many patients might be unemployed, receiving benefits or both other definitions on restricted financial means). Interestingly in 2020 survey, the immunosuppressant medications and the biological agents did not show any significant differences in patients' QoL between the BS groups who used these medications and those who did not. However, steroids or chemotherapy medications (such as Chlorambucil, Methotrexate, and Cyclophosphamide) revealed significant lower QoL in patients taking these medications. Our UK Behçet's treatment pathway used by all Centres broadly follows the European League Against Rheumatism (EULAR) recommendations for the management of Behçet's, 2018 (16, 17).

Smoking cessation and minimising the use of cannabis are significant lifestyle interventions needed to reduce the negative impact on BS patients' QoL and improve the disease outcome experience of patients. Behçet's centres team partners with smoking cessation services for prompt referral and successful intervention.

Patients with low education attainment, have a lower QoL compared with patients with higher education attainment. Higher levels of education attainment might reflect a better ability to navigate the NHS and support services, greater understanding, and compliance with management strategies. In addition, patients who were in full or part-time employment have better QoL than nearly 125 patients who were retired or unemployed because of BS.

Behçet's symptoms patients who are incapacitated and unemployed should have access to support and benefit schemes. However, 2014 and 2020 survey findings showed that QoL was lower in patients who claimed/or received benefits compared to patients who did not.

The last 18 months has seen a significant change in employment due to the SARS-CoV-2 pandemic and a follow up study to review the impact of lock downs and furloughed employment of BS patients will be undertaken.

Limitation of studies carried out in 2009, 2014, and 2020 on HRQoL did not include the BS cardiovascular symptoms. We also did not account for changes to the processing for claiming benefits between, 2009, 2014, and 2020. In addition, patients' disease activity which would impacts on QoL was not included in this survey.

Additionally, these studies were retrospective and comparative cross-sectional studies of the same cohort of BS patients over time. It will be more effective if the studies with continued longitudinal studies.

## Conclusion

In this study, 10 BS symptoms accounted for a 14% variation in patients' QoL. The symptoms that negatively impacts the HRQoL are arthropathy, headache and “other neurological” problems, pathergy reaction, stomach symptoms, and skin lesions.

Behçet's symptoms patients who are in stable marriages, have higher levels of education, practise healthy lifestyle and daily exercise, and work in a supportive employment environment reported better QoL. However, the severity of the symptoms impacts negatively on the ability of BS patients to remain in employment. Psychological support is crucial for the patients. At present added socioeconomic assistance and a dedicated support worker funded through Behçet's Society; Behçet's Patient Support is present in all three the Behçet's Centres. We need to develop strategies to assist patients to return to or, regain financial independence and self-sufficiency through rewarding full or part-time employment. Better understanding of these complex interrelated factors will help BS patients sustain better QoL.

## Data availability statement

The data will be shared on reasonable request to the corresponding authors.

## Ethics statement

Ethical approval was obtained from the Queen Mary Research Ethics Committee; City Research Ethical Committee (COREC) approved study Immune-regulation at the mucosal barrier (P/03/122) at Barts Health NHS Trust in full compliance with the Helsinki Declaration. The patients/participants provided their written informed consent to participate in this study.

## Author contributions

AS: data formatting, data analysis, drafting paper, and reviewed draft. JM: assisted in drafting questions, computerised questions, and reviewed draft. DO: assisted in drafting questions, formatting data, and reviewed draft. LB and BG: critically reviewed and added to paper drafting. FF: planned and oversaw all components of paper. All authors contributed to the article and approved the submitted version.

## Funding

This study was supported by the Behçet's Centre.

## Conflict of interest

The authors declare that the research was conducted in the absence of any commercial or financial relationships that could be construed as a potential conflict of interest.

## Publisher's note

All claims expressed in this article are solely those of the authors and do not necessarily represent those of their affiliated organizations, or those of the publisher, the editors and the reviewers. Any product that may be evaluated in this article, or claim that may be made by its manufacturer, is not guaranteed or endorsed by the publisher.
