# Supplementary figures and images for: The impact of multifactorial factors on the Quality of Life of Behçet's patients over 10 years
Source: Front Med (Lausanne). 2022 Dec 5;9:996571. doi: 10.3389/fmed.2022.996571 (PMC9760941; doi:10.3389/fmed.2022.996571)

Supplementary Materials


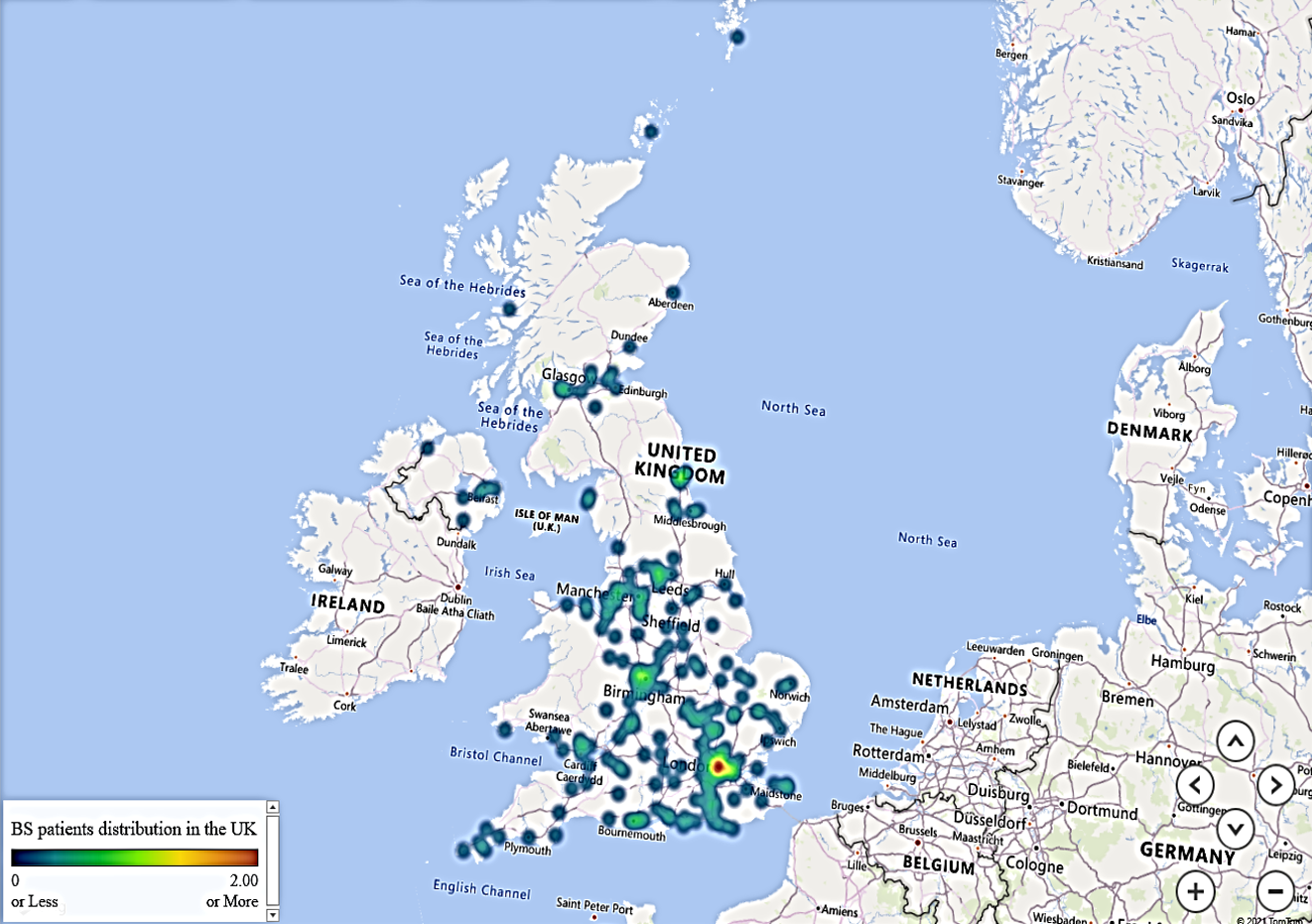


Figure S1: The geographic distribution of 2020 BS patient’s cohort (UK)

Supplement: Supplementary file 1 [file Data_Sheet_1.docx]
