# Supplementary material for: The impact of multifactorial factors on the Quality of Life of Behçet's patients over 10 years
Source: Front Med (Lausanne). 2022 Dec 5;9:996571. doi: 10.3389/fmed.2022.996571 (PMC9760941; doi:10.3389/fmed.2022.996571)
Supplement: Supplementary file 2 [file Data_Sheet_2.docx]

Supplementary Materials


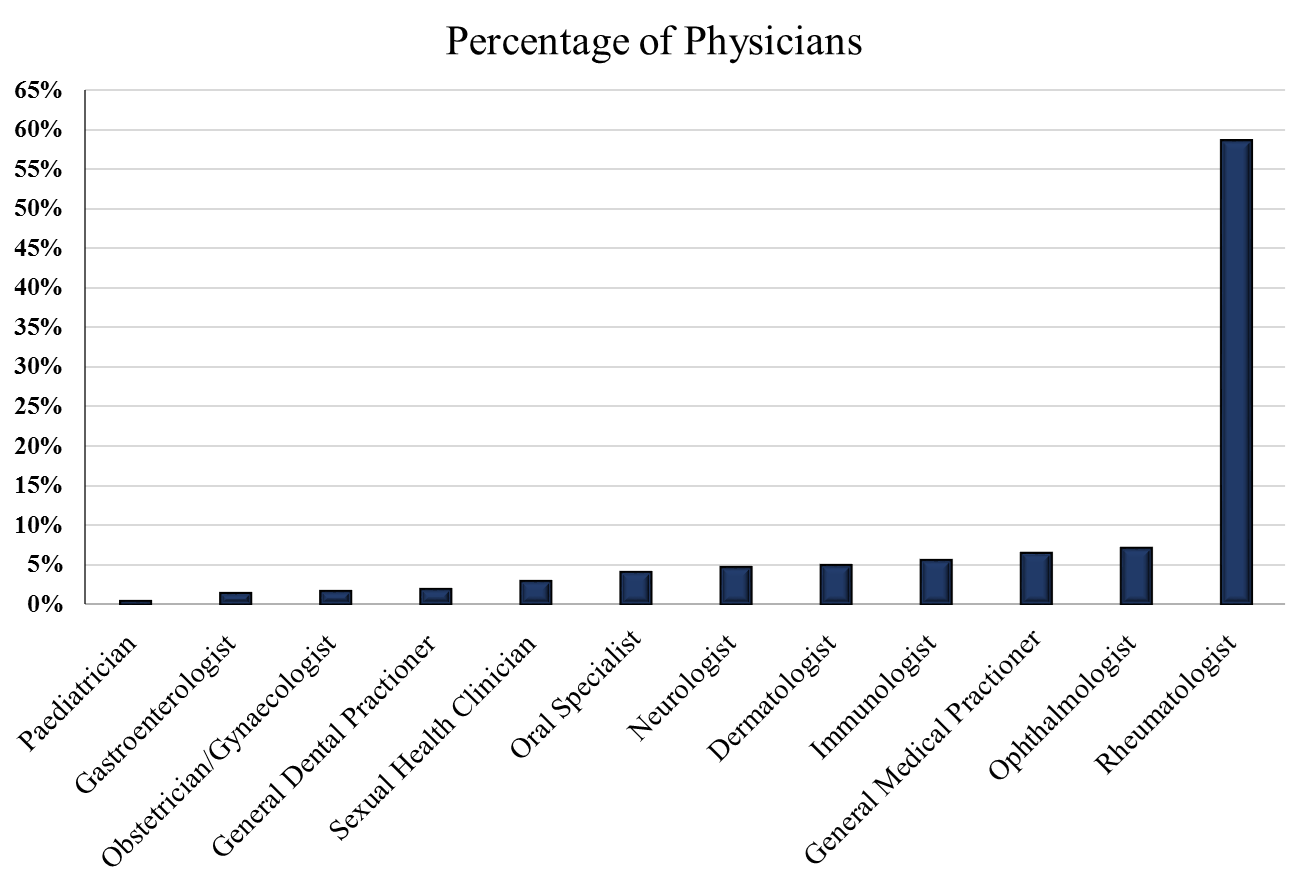


Figure S2: The percentage of physicians who made the initial diagnosis and referred patients to BS centres.
